# Comparison between the areas of scarred and active toxoplasmic retinochoroiditis

**DOI:** 10.1038/s41433-020-01290-3

**Published:** 2020-11-24

**Authors:** Pierre Duraffour, Chadi Mehanna, Florence Hoogewoud, Arnaud Touboul, Dominique Monnet, Antoine P. Brézin

**Affiliations:** 1 Service d’ophtalmologie, Hôpital Cochin, Assistance Publique – Hôpitaux de Paris, Paris, France; 2 Université de Paris, Paris, France; 3 Département de biostatistiques, Hôpital Necker-Enfants Malades, Assistance Publique – Hôpitaux de Paris, Paris, France

**Keywords:** Parasitic infection, Retinal diseases, Uveal diseases

## Abstract

**Background/objectives:**

To assess the ratio of scarred/active areas of fundus lesions in patients with presumed ocular toxoplasmosis.

**Subjects/methods:**

Retrospective monocentric study of patients with presumed ocular toxoplasmosis seen between May 2004 and February 2018. Patients with a positive anti-Toxoplasma serology presenting characteristic fundus lesions. Cases with images of both baseline active and scarred lesions of the fundus were included. The borders of each active or scarred lesion were delineated on colour photographs by two independent observers and the area of the lesions was calculated using Digimizer 4.2.2 (MedCalc Software, Ostend, Belgium). The interobserver variability of the measures was recorded and their means were used for further calculations. To study the ratio of the area of scarred retinochoroiditis over the area of the baseline active lesion (*R*).

**Results:**

A total of 171 cases (83 males, 88 females) with a mean age of 31.6 ± 13.8 years were included. The average areas of active and scarred retinochoroiditis were, respectively, 1.32 ± 1.59 and 1.79 ± 2.36 optic disc area. The average ratio between scarred and active areas of retinochoroiditis was 1.36 [range 0.54–2.18]. The administration of a systemic treatment [*R* = 1.25, *p* = 0.003], the absence of a pre-existing scar [*R* = 1.05, *p* < 0.001] and a peripapillary location of the lesion [*R* = 0.85, *p* < 0.001] were each significantly associated with smaller scarred/active area ratios.

**Conclusions:**

We assessed in a standardized manner the ratio of scarred/active areas of toxoplasmic lesions and showed that the area of scarred lesions was on average slightly larger than the area of active retinochoroiditis.

## Introduction

Ocular toxoplasmosis is the most common cause of infectious uveitis [1, 2]. Toxoplasmic lesions of the fundus appear as active whitish areas of retinochoroiditis or as inactive pigmented and/or atrophic scars. The manifestations can be the consequence of a congenital or an acquired infection. The localization of the lesion is the main factor affecting the patients’ visual outcome [3–5]. Lesions in the macular area can result in decreased visual acuity, while peripheral lesions usually result in little or no visual impairment. Active retinochoroiditis is often adjacent to a pigmented scar. Symptoms of active toxoplasmic retinochoroiditis are due to vitritis and/or to the scotoma related to the affected area [3, 5, 6]. An immunologic deficiency can be a trigger for the reactivation of ocular toxoplasmosis but other factors are still unidentified. Active ocular toxoplasmosis usually develops into a scarred lesion within a few weeks but the larger the lesions, the longer the process [7]. There is no consensus regarding the treatment of ocular toxoplasmosis [8–26]. A report by the American Academy of Ophthalmology concluded that there is a lack of level I evidence to support the efficacy of routine antibiotic or corticosteroid treatment for acute toxoplasmic retinochoroiditis [26]. Studies assessing the effect of treatment for ocular toxoplasmosis can be based on various parameters, including visual acuity, the duration of the inflammation and the recurrence rate [4, 7, 10, 12–16, 18–25, 27, 28]. Overall, in these studies, the ratio of the scarred/active areas of the lesions is one of the least used parameters. The lack of a consensual method to assess this ratio is perhaps one of the reasons explaining this relative lack of data. Yet, the area of a lesion directly affects the vision when located near the macula [3, 4, 6]. The goal of our study was therefore to assess the ratio of the scarred/active area of retinochoroiditis in ocular toxoplasmosis.

## Methods

### Study design and patients

This was a retrospective study of patients seen in the department of Ophthalmology at Cochin University Hospital between May 2004 and February 2018. Patients with a diagnosis of presumed ocular toxoplasmosis were included. The diagnosis was based on the presence of a characteristic fundus lesion and a positive Toxoplasma serology. The origin of the infection was categorized as congenital or acquired when known and the cases which occurred in the context of an immunologic deficiency were recorded. An active lesion was diagnosed in the presence of a whitish focus of retinochoroiditis, without well-limited borders, frequently adjacent to a pigmented and/or atrophic scar. For the purpose of our study, cases were included when a baseline fundus photograph of the active lesion was available, as well as a fundus photograph of the scarred lesion. The study protocol was approved by the ethics committee of the Société Française d’Ophtalmologie (IRB 00008855).

### Assessment of lesions

**Fig. 1:**
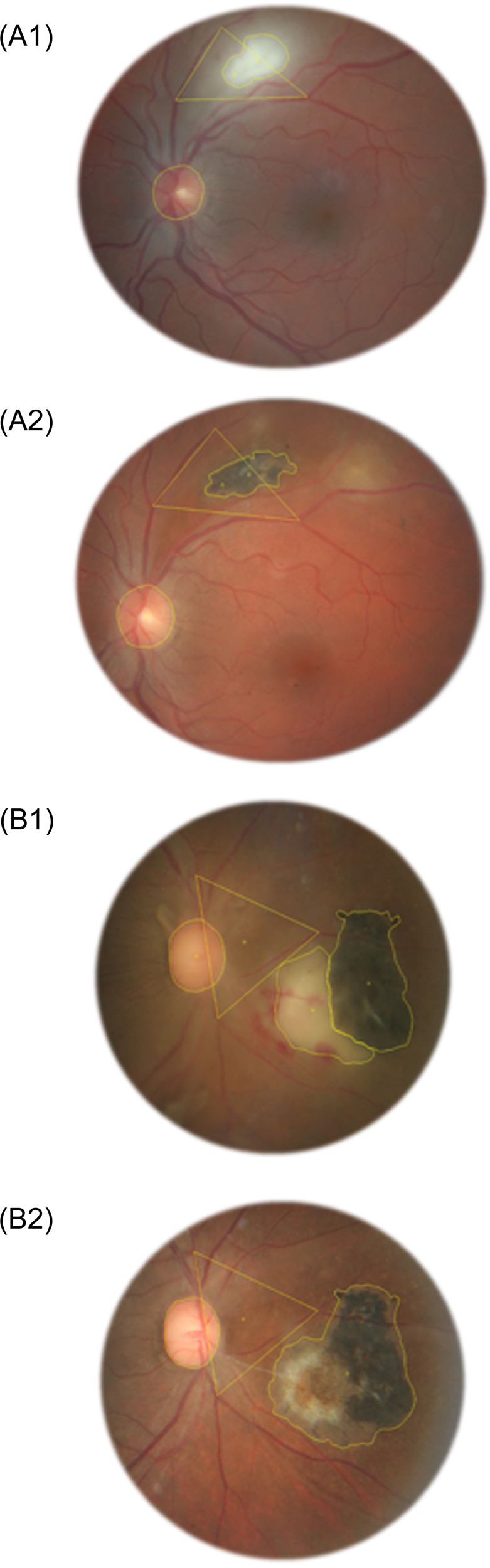
Assessment of the area of the active and scarred lesions. For every case, the area of the lesions both at the active and the scarred stages was measured using the Digimizer4.2.2 software. The optic disc was delineated on every picture as a reference unit to assess the size of the lesion. A reference area based on the marking of vascular bifurcations was used for comparisons between photographs at the active and scarred stages. **A1** A 33-year-old patient presented with active retinochoroiditis. The area of the active retinochoroiditis was delineated on the baseline fundus photograph. **A2** The fundus was photographed again at the scarred stage. The scarred area of retinochoroiditis was also delineated. **B1** A 23-year-old patient presented with an active retinochoroiditis contiguous to a retinochoroidal scar. Both the active lesion and the preexisting scar were delineated. **B2** The fundus was photographed again at the scarred stage. The entire scarred area of retinochoroiditis was delineated. The area of scarred retinochoroiditis linked to this episode was measured as the entire area of the scar minus the area of the preexisting scar.

Fundus photographs were taken using a 60° angle wide camera or an ultra-wide-field Scanning Laser Ophthalmoscopy (SLO). For each case, the active and the scarred lesions were compared using the same imaging technique. The contours of the scarred lesions were delineated based on their colour, with the scar limit positioned at the border of the pigmented or atrophic area. When the active lesion was contiguous to a retinochoroidal scar, the two areas were delineated distinctively. Each lesion’s surface was calculated using the Digimizer 4.2.2 software (MedCalc Software, Ostend, Belgium) (Fig. 1). Two observers (PD and AT) independently performed these assessments of the areas of the active and scarred lesions. Additionally, reference areas were calculated by the delineation of geometrical figures using vascular bifurcations as fixed edges (Fig. 1). The patient’s optic disc area was used as a reference surface unit allowing comparisons between the surface of active and scarred retinochoroiditis. When the measure of the area by one of the observers was at least four times larger than the other observer’s, a joint reassessment of the lesion was performed and was used for further calculations. The localization of the retinochoroiditis was categorized as zones 1, 2 or 3 according to their distance from the macula and optic disc [29]. In addition, lesions immediately contiguous to the optic disc were specifically categorized. The antiparasitic and corticosteroid treatments prescribed between the time of the initial and final fundus photographs were recorded. The delay between the first symptoms reported by the patients and the baseline photograph, as well as the time interval between the baseline and final fundus photographs were analysed.

### Statistical analysis

Variables were presented as means ± standard error, their distribution was assessed using the Lilliefors test, and a logarithmic transformation was applied to non-normal variables. Bland–Altman graphs and intraclass correlation coefficients (ICCs) were used to assess inter-observer agreement in area evaluation, and the mean between the two observers was used for subsequent analysis. Pearson’s correlation coefficient was used for normal variables and Spearman’s rank correlation coefficient was used otherwise. The ratio (*R*) of scarred/baseline active area was calculated, and its correlation to different predictive variables was evaluated using the Wilcoxon test. After logarithmic transformation of predictive variables, univariate and multivariate linear regression models were fitted using forward variable selection. Best fit models were chosen according to the smallest Akaike Information Criterion (AIC) and preferably the smallest Bayesian Information Criterion (BIC). All the statistical analyses were realized using the statistical software R version 3.5.0 (R Core Team (2018)).

## Results

During the study period, we identified 171 cases of ocular toxoplasmosis which met our inclusion criteria. The patient demographics and the main characteristics of the lesions are shown in Table 1. There were 83 males and 88 females with a mean age of 31.6 ± 13.8 years. The infection was from a known congenital origin in 10 (5.8%) patients, from a known acquired origin in 8 (4%) patients, and otherwise from an unknown mode. Among our patients, 116 had one or more scarred lesion on the baseline image. Of the 55 patients without a pre-existing scar, none had a known congenital infection and 6 had a positive titre of anti-Toxoplasma immunoglobulin M. An immunologic deficiency was recorded in 10 (5.8%) patients. Among 342 measures of the toxoplasmic lesions (171 active and 171 scarred) performed by each examiner, 18 required a joint reassessment. Fundus photographs were taken using a 60° angle wide camera for 160 patients and using ultra-wide-field SLO for 11 patients. The average area of these 18 lesions was 0.55 optic disc area and 12 had an area smaller than 1/3 of the optic disc area. Interobserver agreement was measured for both active and non-active lesions. A logarithmic transformation was used to normalize variable distribution. The homogeneity of variances was preserved. The ICCs were 0.92 [0.90; 0.94] and 0.95 [0.93; 0.96] for the active and the non-active lesions, respectively. The Bland–Altman graphs showed a homogeneous distribution of the differences between the two observers relative to the average value. No significant outliers were recorded. The median delay between baseline and final fundus photographs was 85 days (interquartile range [38; 361]). Among the study patients, 139 (81%) were prescribed an oral antiparasitic treatment, 123 (72%) of whom were also treated by systemic corticosteroids. The most frequently prescribed treatment was the combination of pyrimethamine and azithromycin, used in 133 (77.8%) cases. None of the patients was treated by corticosteroids alone. The average duration of the antiparasitic treatment was 26.80 ± 11.90 days. Among the 19 untreated patients, none had a lesion located in zone 1.

**Table 1:** Patients demographics and characteristics of the fundus lesions.

Patient demographics	Values
Total number of patients	171
Men	83 (48.5%)
Women	88 (51.5%)
Mean age (years)	31.6 ± 13.8
Time interval	
Mean time interval between the first symptoms and the baseline image (days)	13.43 ± 14.96
Median time interval between baseline and scarred lesion imaging (days)	85 [range 38; 361]
Lesion location	
Zone 1 non adjacent to the optic disc	60 (35.1%)
Zone 1 adjacent to the optic disc	19 (11.1%)
Zone 2	81 (47.4%)
Zone 3	11 (6.4%)
Lesion presentation	
Presence of one or more scarred lesions on the baseline image*	116 (67.8%)
Active lesion only	55 (32.2%)

*Whether or not the scar was contiguous to the active lesion.

**Fig. 2:**
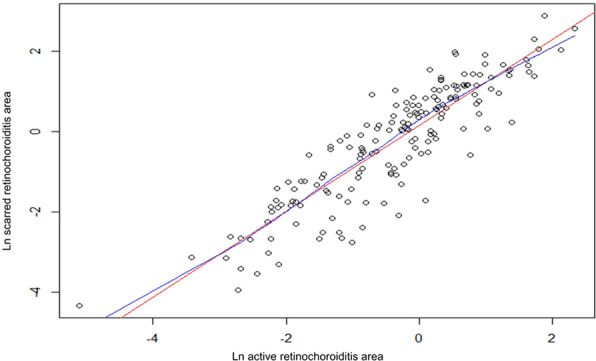
Areas of retinochoroiditis at the active and the scarred stages. Correlations between the areas at the active (*x*-axis) and scarred stages (*y*-axis) were assessed using a logarithmic transformation of the measures. Linear (in red) and nonlinear (“LOWESS”) (in blue) regression lines are shown.

The average areas of active and scarred retinochoroiditis were, respectively, 1.32 ± 1.59 and 1.79 ± 2.36 optic disc area. There was a significant linear correlation between the active retinochoroiditis area and the scarred retinochoroiditis area after logarithmic transformation of values; the linear correlation coefficient of Pearson was 0.89 [0.85; 0.92] (Fig. 2). Globally, for the 171 patients, the retinochoroiditis lesion area slightly increased between the active and scarred stages, with an average ratio of 1.36 [0.54–2.18]. The results of univariate analyses are shown in Table 2. The 139 patients who received a systemic antiparasitic treatment (*R* = 1.25 [0.48–2.03]) had a smaller average ratio than the 19 untreated patients (*R* = 1.85 [1.01–2.69]) (Wilcoxon test, *p* = 0.003). The 123 patients who were treated by systemic corticosteroids and antiparasitic treatment (*R* = 1.20 [0.49–1.90]) had a smaller average ratio than the 48 patients who were not treated by systemic corticosteroids (*R* = 1.79 [0.85–2.72]) (Wilcoxon test, *p* = 0.001). The 19 patients with a lesion adjacent to the optic disc (*R* = 0.85 [range 0.30–1.40]) had a smaller average ratio than the 152 other patients (*R* = 1.43 [range 0.60–2.25]) (Wilcoxon test, *p* = 0.001). The 55 patients without scarred lesions on their baseline photograph (*R* = 1.05 [range 0.24–1.86]) had a smaller average ratio than the 116 patients with at least one scarred lesion at baseline, whether or not the scar was contiguous to the active lesion (*R* = 1.51 [range 0.73–2.30]) (Wilcoxon test, *p* = 0.001). After univariate analysis, significant factors were selected and included in a multivariate regression model. The best fit model included the following significant variables: administration of a systemic treatment (*p* = 0.005), pre-existing scarred lesion whether or not contiguous to the active lesion (*p* = 0.004) and peripapillary location (*p* = 0.028). In this model, for treated patients, corticosteroids had no significant effect on the mean scarred/active ratio and could be considered as a confounding factor with antiparasitic treatment.

**Table 2:** Analyses of the ratio of scarred/active area of chorioretinitis according to the patient demographics and characteristics of the lesions.

Analysis criteria	Subset 1		Subset 2		Wilcoxon’s Test
Gender	Male (*n* = 83)	1.393 ± 0.844	Female (*n* = 88)	1.335 ± 0.797	*p* = 0.795
Infection modalities	Known congenital toxoplasmosis (*n* = 10)	1.291 ± 0.704	Other or unknown (*n* = 161)	1.367 ± 0.827	*p* = 0.790
Immune status	Immunodeficient (*n* = 10)	1.473 ± 0.9	Immunocompetent (*n* = 161)	1.356 ± 0.815	*p* = 0.841
Preexisting scar	Present (*n* = 116)	1.512 ± 0.783	Absent (*n* = 55) (including 6 patients with positive IgM titres).	1.048 ± 0.81	*p* = 0.00003
Location of the lesions	Adjacent to the disc (*n* = 19)	0.846 ± 0.549	Non adjacent to the disc (*n* = 152)	1.428 ± 0.826	*p* = 0.0006
	Zone 1 (*n* = 79)	1.190 ± 0.667	Zone 2 or 3 (*n* = 92)	1.511 ± 0.906	*p* = 0.0611
Antiparasitic treatment	Treated (*n* = 139)	1.255 ± 0.773	Untreated (*n* = 19)	1.853 ± 0.840	*p* = 0.0026
Corticosteroids	With corticosteroids (*n* = 123)	1.198 ± 0.704	Without corticosteroids (*n* = 48)	1.785 ± 0.938	*p* = 0.0008

Neither the time interval between the baseline and final fundus photographs (Spearman coefficient 0.04 (*p* = 0.58)) nor the delay between the first reported symptoms and the baseline photograph (Spearman coefficient 0.05 (*p* = 0.52)) had a significant influence on the scarred/active ratio.

## Discussion

Our results show on average a slight increase in the size of the scarred area of ocular toxoplasmosis over the area of active retinochoroiditis. In contrast with the relatively large number of series of patients with ocular toxoplasmosis, we have identified only seven reports in ophthalmic literature which included the area of the lesions as an outcome measure [7, 12–17]. They all included a smaller number of cases than in our study. This relative lack of data can have several causes, such as missing images once the lesions have become inactive. However, one of the main difficulties for the study of the course of toxoplasmic lesions is the lack of a standardized method to delineate the border of the affected area, especially at the active stage. Most of our measures were based on 60° standard images, while only a few cases used ultra-wide-field SLO. Hence, we were unable to compare the reproducibility of these imaging modalities. Oedema around active lesions is a common manifestation as can be seen on fluorescein angiograms or on OCT imaging. Hence, delineating the exact limit of the area of active toxoplasmic retinochoroiditis can be challenging. In their study, Soheilian et al. showed an example of the delineation of an active lesion that clearly included the area of oedema beyond the active lesion [16]. Similarly, Balaskas et al. used late phase images of fluorescein angiograms to delineate the lesions and included the area of leakage beyond the limit of the lesion as seen on the clinical examination of the fundus [14]. More recently, Lashay et al. used autofluorescence to analyse the lesions, which can also result in a larger assessment of their size [12]. To overcome the difficulty of precisely assessing the boundary of the lesions, we used a delineation method based on colour fundus photographs and we set the active side of the border at the limit of the whitish area. Using this method, the areas of the lesions delineated by two independent observers were consistently similar. Discrepancies between the observers occurred mainly for the smallest lesions. As the area is a function of the square of the diameter of a lesion, the delineation of lesions smaller than ½ disc diameter resulted in the least concordance between the observers. These discrepancies were resolved by a joint reassessment of the lesions’ areas. In our study, the areas of the delineated lesions were computed with the Digimizer software as used by Lashay et al., whereas in several other reports the areas were assessed on manual drawings of the fundus [12]. The time interval between the analysis of the active and the scarred areas can be another factor resulting in variable results. In three reports, a fixed time interval of 6 weeks separated the assessment of the area of retinochoroiditis [7, 16, 17]. Among cases from these reports, the final assessment of the lesions’ size might have been performed before the lesions reached the fully scarred stage. Yet, large lesions can take longer to reach a completely scarred stage [5]. Therefore, to assess the area of the toxoplasmic scar, we used photographs taken as late as needed, when the lesions were fully inactive. However, our study methods did not allow us to measure the kinetics of the scarring process. We observed that the lesions adjacent to the disc had a smaller than average ratio of scarred/active area of toxoplasmic retinochoroiditis. This could be partly explained by the boundary of the disc limiting the extension of the lesion during the course of the scarring process. When active lesions were seen in addition to preexisting scars, a greater ratio was observed.

Analyzing the effect of treatment methods on the ratio of scarred/active area of toxoplasmic retinochoroiditis was not our primary objective. Rothova et al. compared three treatment regimens—combinations of pyrimethamine + sulfadiazine, clindamycin + sulfadiazine and trimethoprim–sulfamethoxazole—with lack of treatment for peripheral lesions [7]. After 42 days, a reduction in the area of the toxoplasmic lesions was observed in 49% of the pyrimethamine-treated patients compared to only 20% of the untreated ones [7]. Yet, because wide-field imaging was not available at the time of their study, the authors used manual drawings to assess peripheral lesions [7]. Our study included treated as well as untreated patients and, as in other reports, the untreated lesions were localized in the periphery, while zone 1 lesions were treated [7]. A combination of pyrimethamine and azithromycin was the most commonly used antitoxoplasmic treatment in our series, but because of the dissimilar location of treated and untreated lesions we do not believe that meaningful conclusions regarding the effect of treatment on the size of lesions can be drawn. Corticosteroids were prescribed in 123 of 171 cases, but similarly, we cannot evaluate their effect on the ratio of scarred/active area of toxoplasmic lesion. This is in line with a recent Cochrane review which could not find evidence for or against the role of corticosteroids in the management of ocular toxoplasmosis [30].

Based on a standardized assessment method, our results showed that overall toxoplasmic scars were larger than the area of active retinochoroiditis. Several recent reviews, by the American Academy of Ophthalmology and the Cochrane organization among others, highlighted the need to gather more data to support therapeutic interventions in active toxoplasmic retinochoroiditis [10, 26, 30]. The results of our study could be used as a benchmark for future treatment trials aiming at limiting the size of toxoplasmic lesions.

## Summary

### What was known before

- Lesions of ocular toxoplasmosis evolve from an active to a quiescent stage.
- The lesions’ final size is a key element of the prognosis.

### What this study adds

- Standardized photographic assessments showed that the area of scarred toxoplasmic lesions was on average 1.36 larger than the area of active retinochoroiditis.

## Supplementary information

AT - Asseessment of the areas

PD - Assessment of the areas

## Data Availability

All data relevant to the study are uploaded as supplementary information.
